# Substantial diagnostic impact of blood culture independent molecular methods in bloodstream infections: Superior performance of PCR/ESI-MS

**DOI:** 10.1038/s41598-018-34298-7

**Published:** 2018-10-30

**Authors:** Athanasios Makristathis, Nicole Harrison, Franz Ratzinger, Manuel Kussmann, Brigitte Selitsch, Christina Forstner, Alexander M. Hirschl, Heinz Burgmann

**Affiliations:** 1 Division of Clinical Microbiology, Department of Laboratory Medicine, Medical University Vienna, Vienna, Austria; 2 Division of Infectious Diseases and Tropical Medicine, Department of Medicine I, Medical University Vienna, Vienna, Austria; 3 Division of Medical-Chemical Laboratory Diagnostics, Department of Laboratory Medicine, Medical University Vienna, Vienna, Austria; 4 Centre of Infectious Diseases and Infection Control, Jena University Hospital, Jena, Germany

## Abstract

This study analyzed the performance of different molecular technologies along with blood culture (BC) in the diagnosis of bloodstream infections (BSI) in patients from internal medicine wards - including intensive care units (ICUs) - and the emergency room. Patients with systemic inflammatory response syndrome were prospectively included. BCs and EDTA whole blood were obtained simultaneously. The latter was analyzed by PCR combined with electrospray ionization mass spectrometry (PCR/ESI-MS; IRIDICA BAC BSI assay, Abbott) and by SeptiFast (Roche). Cases were classified as BSI according to adapted European Centre for Disease Prevention and Control criteria. Out of 462 analyzed episodes, 193 with valid test results fulfilled the inclusion criteria and were further evaluated. Sixty-nine (35.8%) were classified as BSI. PCR/ESI-MS showed a significantly better overall performance than BC (p = 0.004) or SeptiFast (p = 0.034). Only in patients from the ICU the performance of SeptiFast was comparable to that of PCR/ESI-MS. Mainly due to the negative effect of antimicrobial pre-treatment on BC results, the cumulative performance of each of the molecular tests with BC was significantly higher than that of BC alone (p < 0.001). SeptiFast and in particular the broad-range pathogen detection system PCR/ESI-MS proved to be an essential addition to BC-based diagnostics in BSI.

## Introduction

Sepsis is a common cause of morbidity and mortality^1,2^. Following the SEPSIS 1 definition of the ACCP/SCCM consensus scheme, sepsis is considered as systemic inflammatory response syndrome (SIRS) due to a highly suspected or documented infection^3,4^. However, apart from bloodstream infections (BSI), SIRS is also prevalent in other critically ill patients and in post-surgical conditions^5,6^. Given that no single bedside test provides clinical evidence of sepsis, in order to be able to recognize an infectious condition behind SIRS, the identification of the causative pathogen(s) during microbiological diagnostics is particularly important. Currently, the latter is based mainly on blood culture (BC)^7–9^. However, this method is often associated with low clinical sensitivity and a considerable time delay. The lack or delay of microbiological findings may result in wrong therapeutic decisions including inappropriate use of antimicrobial therapy. This may not only negatively impact the clinical outcome for the infected patient, but is also associated with the increase of antibiotic resistance and costs^10–12^.

As an alternative to conventional BC techniques, molecular techniques for detection of microorganisms from whole blood have been made available^13–22^. These technologies have potential advantages by decreasing the time required for a result, reducing inhibitory effects caused by prior use of antibiotics, and improving the detection of slow growing or fastidious organisms. Currently the most well-established, commercially available molecular technique is SeptiFast^®^ (SF; Roche Diagnostics, Rotkreuz, Switzerland), a real-time PCR based test allowing for the detection of pathogens which - according to the manufacturer - are responsible for approximately 90% of sepsis episodes. However, less common sepsis pathogens, which are not included in the respective panel, cannot be detected either by this or other molecular tests targeting a limited spectrum of microorganisms. Consequently, the introduction of molecular methods allowing for the broad-range detection of pathogens in whole blood would be preferable.

During recent years, a promising technology based on PCR followed by electrospray ionization mass spectrometry (PCR/ESI-MS) has been introduced. This technique, which allows for the comprehensive detection and quantitation of clinically relevant bacteria and yeasts in sterile tissues and fluids including whole blood^23–26^, has then been approved for *in vitro* diagnostic use (IRIDICA system, Abbott Diagnostics, Lake Forest, IL, USA).

The aim of the present study was to evaluate the accordance of PCR/ESI-MS (IRIDICA BAC BSI assay), SF and BC with an adapted BSI diagnosis based on the European Centre for Disease Prevention and Control (ECDC) criteria for the point prevalence survey of healthcare-associated infections^27^. The impact of prior antimicrobial treatment, intensive care, neutropenia and infection focus on the performance of each of these three tests has been assessed.

## Results

### Study cases

From a total of 462 episodes with a suspected BSI, in which BC and both molecular tests have been performed, only 201 cases fulfilled the requirement of at least two SIRS criteria, and have therefore been included in the study and were further analyzed. Eight of these cases (4%) were excluded from statistical evaluations due to an invalid analysis by PCR/ESI-MS as reported by the system’s software. Out of the eight cases, in six both BC and SF were negative, in another BC was negative and SF was inhibited; however, in one of these cases both tests were positive with *Streptococcus pneumoniae*. Of the remaining 193 study cases, the comparison of demographic and clinical data between those classified as BSI (n = 69) and those classified as non-BSI (n = 124) did not reveal any considerable differences (Table 1). Most noticeable was the significantly higher occurrence of central venous catheters in BSI than in non-BSI patients (47.8% vs. 24.2%, p = 0.001).

**Table 1:** Demographic and clinical data of study patients.

Parameter	Non-BSI (n = 124, 64.2%)	BSI (n = 69, 35.8%)	p-value
**Nominal/Ordinal**	**Count (Percentage)**		**chi-square-test**
Male:Female	83:41	45:24	0.874
ER:ICU:SC:SC o/h	6:16:57:45	10:9:30:20	0.125
Neoplasia	55 (44.7%)	27 (39.7%)	0.544
Antibiotics naivety	53 (42.7%)	28 (40.6%)	0.879
Intubation	13 (10.5%)	12 (17.4%)	0.180
Urinary catheter	21 (17.2%)	21 (30.4%)	0.045
Central venous catheter	30 (24.2%)	33 (47.8%)	0.001^a^
Other catheter types	20 (16.1%)	14 (20.3%)	0.555
Chills^b^	51 (41.1%)	34 (49.3%)	0.293
**Numeric**	**Median (Quartile 1- Quartile 3)**		**Mann-Whitney-U-test**
Age	57.0 (37.0–69.0)	56.0 (48.0–68.0)	0.923
BMI	25.0 (19.5–28.0)	23.0 (18.0–28.0)	0.347
Heart beat rate	100.0 (92.0–106.0)	100.0 (93.0–113.5)	0.115
Body temperature	38.3 (38.1–38.9)	38.4 (38.1–39.0)	0.398
MAP	84.7 (75.9–94.7)	81.8 (70.0–91.7)	0.088
Respiration rate	24.0 (23.0–28.0)	24.0 (21.0–28.0)	0.438
Oxygen saturation	97.0 (94.0–98.0)	96.0 (91.0–97.0)	0.123
CRP	9.6 (5.4–18.6)	12.1 (5.7–22.5)	0.564
Neutrophils (%)	74.3 (49.3–84.8)	81.2 (60.7–88.2)	0.047
Lymphocytes (%)	14.6 (7.4–27.5)	7.5 (4.9–23.4)	0.029
N/L ratio	5.1 (2.4–11.3)	10.8 (2.8–17.4)	0.042
White blood cell count	7.8 (2.1–13.1)	9.5 (1.5–14.5)	0.805

BSI = bloodstream infection; ER = emergency room; ICU = intensive care unit; SC = standard care wards; SC o/h = standard care wards oncology/hematology; ^a^significant after applying the Bonferroni-Holm correction; ^b^in 45 cases (non-BSI: n = 27; BSI: n = 18) occurrence of chills was not assessable; BMI = body mass index; MAP = mean arterial pressure; CRP = C-reactive protein; N/L Ratio = neutrophils/lymphocytes ratio.

### Potential pathogens and contaminants

**Figure 1:**
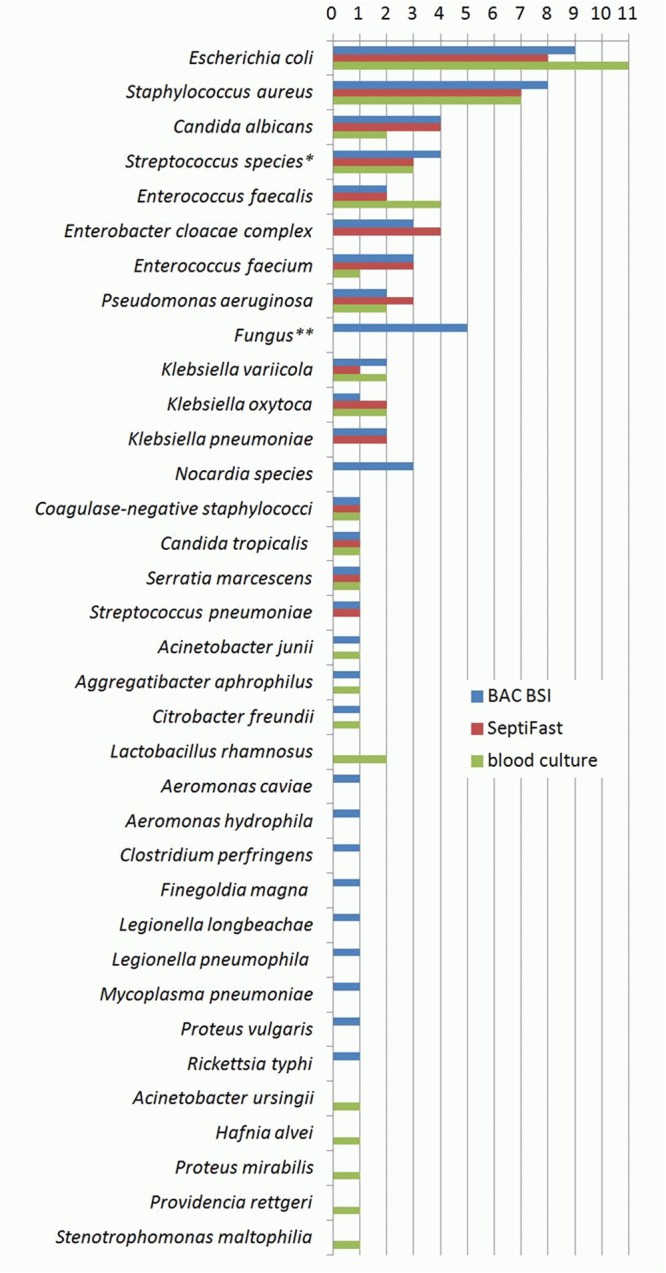
Overall distribution of potential pathogens. *Out of three cases with *Streptococcus* species detected by SeptiFast, two corresponded to *Streptococcus mitis/oralis* and one to *Streptococcus agalactiae* as shown by both BAC BSI and blood culture; in another case *Streptococcus agalactiae* was detected only by BAC BSI. **Fungus other than *Candica* species was detected, but identification could not be provided.

The overall distribution of potential pathogens detected during the study by each of the three tests is shown in Fig. 1; microorganisms classified as contaminants are not included. Contamination was found by BC and/or by PCR/ESI-MS in 13 non-BSI cases. Of those, in eight only BC was positive (4 × *Staphylococcus epidermidis*, 2 × *Propionibacterium acnes*, 1 × *Staphylococcus hominis*, 1 × *Staphylococcus haemolyticus* – in each case only one out of four to six bottles of a single blood sample within 48 h), in four only PCR/ESI-MS was positive (3 × *Propionibacterium acnes*, 1 × *Staphylococcus lugdunensis*), and in one both tests were positive with a different microorganism (*Rothia mucilaginosa* by BC in a single bottle and *Propionibacterium acnes* by PCR/ESI-MS).

### Overall test performance

The overall agreement between the three tests was 78.8%; the highest agreement of 84.5% was found between the two molecular tests followed by 81.9% between BC and PCR/ESI-MS and 79.8% between BC and SF. In cases of a positive result by more than one test, agreement was regarded if the same pathogen has been identified by the different tests. In three BSI cases *Streptococcus* species detected by SF corresponded to *Streptococcus mitis/oralis* in two cases and *Streptococcus agalactiae* in one case by both PCR/ESI-MS and BC. In a further case of BSI due to a central venous catheter infection coagulase-negative staphylococci detected by SF corresponded to *Staphylococcus epidermidis* by the other two tests. Because SF does not allow for a more precise identification of streptococci other than *Streptococcus pneumoniae* and staphylococci other than *Staphylococcus aureus*, in these four cases agreement was assumed between the three tests. Furthermore, in one case of *Escherichia coli* by SF and *Escherichia coli* plus *Proteus vulgaris* by PCR/ESI-MS and another case of *Klebsiella pneumoniae/oxytoca* by SF and *Klebsiella oxytoca* plus *Hafnia alvei* in blood culture, the respective tests were considered to be in agreement despite the detection of an additional pathogen by PCR/ESI-MS or BC. The latter may have been due to the fact that *Proteus vulgaris* and *Hafnia alvei* are not included in the SF panel.

In comparison to BC the performance of PCR/ESI-MS was similar to that of SF (Table 2). Although not of statistical significance, the BAC BSI assay showed higher sensitivity but lower specificity than SF. The nine cases of contamination by BC were considered as negative for this analysis. With respect to the four resistance markers determined by BAC BSI, there was total agreement between the results of the molecular test and BC findings. In comparison to BSI diagnosis according to the adapted ECDC criteria as reference, the overall performance of the BAC BSI assay was significantly higher than that of SF (p = 0.034) and in particular than that of BC (p = 0.004). This finding was attributable to the considerably higher sensitivity of PCR/ESI-MS in comparison to the other tests (Table 3). Since it is reasonable to use BC-independent molecular assays in addition to BC and not as stand-alone tests in BSI diagnostics, the cumulative performance characteristics of each of the tests together with BC have been analyzed. The highest performance was achieved by BC combined with PCR/ESI-MS rather than with the SF (p = 0.011, Table 3).

**Table 2:** BAC BSI and SeptiFast in comparison to blood culture.

blood culture	BAC BSI		SeptiFast	
	neg	pos	neg	pos
neg	122	29	140	11
pos	8	34	11	31
**ROC-AUC (95% CI)**	**0.81 (0.75–0.86)**		**0.83 (0.76–0.88)**	
% sensitivity (95% CI)	81.0 (65.9–91.4)		73.8 (58.0–86.1)	
% specificity (95% CI)	80.8 (73.6–86.7)		92.7 (87.3–96.3)	
% PPV (95% CI)	54.0 (45.0–62.7)		73.8 (60.8–83.7)	
% NPV (95% CI)	93.9 (89.0–96.6)		92.7 (88.4–95.5)	

ROC-AUC = receiver operating characteristic-area under the curve; PPV = positive predictive value; NPV = negative predictive value; CI = confidence interval.

**Table 3:** Test performance in comparison to BSI diagnosis according to the adapted ECDC criteria.

BSI	BC		BAC BSI		SeptiFast		BC + BAC BSI		BC + SeptiFast	
	neg	pos	neg	pos	neg	pos	neg	pos	neg	pos
neg	115	9	119	5	124	0	111	13	115	9
pos	27	42	11	58	27	42	3	66	16	53
**ROC-AUC (95% CI)**	**0.77 (0.70–0.83)**		**0.90 (0.85–0.94)** ^a^		**0.80 (0.74–0.86)**		**0.93 (0.88–0.96)** ^b^		**0.85 (0.79–0.90)** ^c^	
% sensitivity (95% CI)	60.9 (48.4–72.4)		84.1 (73.3–91.8)		60.9 (48.4–72.4)		95.7 (87.8–99.1)		76.8 (65.1–86.1)	
% specificity (95% CI)	92.7 (86.7–96.6)		96.0 (90.8–98.7)		100 (97.1–100)		89.5 (82.7–94.3)		92.7 (86.7–96.6)	
% PPV (95% CI)	82.4 (70.8–90.0)		92.1 (83.0–96.5)		100 (−)		83.5 (75.2–89.5)		85.5 (75.6–91.8)	
% NPV (95% CI)	81.0 (76.0–85.2)		91.5 (86.3–94.9)		82.1 (77.4–86.0)		97.4 (92.4–99.1)		87.8 (82.3–91.7)	

BSI = bloodstream infection; ECDC = European Centre for Disease Prevention and Control; BC = blood culture; ROC-AUC = receiver operating characteristic-area under the curve; PPV = positive predictive value; NPV = negative predictive value; CI = confidence interval; ^a^BAC BSI vs. BC: p = 0.004 and BAC BSI vs. SeptiFast: p = 0.034, ^b^BC + BAC BSI vs. BC: p < 0.001 and BC + BAC BSI vs. BC + SeptiFast: p = 0.011, ^c^BC + SeptiFast vs. BC: p < 0.001 as assessed by the Hanley & McNeil test.

### Test performance in association with clinical criteria/the assigning ward

The performance characteristics of each of the tests in association with prior antimicrobial treatment, the assigning ward, or the infection focus, as determined by the use of the ECDC classification criteria (Supplementary Table S1), is shown in Table 4. While the performances of the three tests did not differ in patients without antimicrobial pre-medication, in those with antimicrobial therapy (n = 112, 58%) the diagnostic efficiency of PCR/ESI-MS was significantly higher than that of BC or SF (p < 0.001 and p = 0.028, respectively). With regard to the assigning ward, both SF and in particular PCR/ESI-MS performed significantly better than BC in patients from the ICU (SF vs. BC: p = 0.049; BAC BSI vs. BC: p = 0.007). Furthermore, the performance of PCR/ESI-MS was found to be significantly better than that of BC in patients from standard care oncology and hematology wards. Overall, 45 of the study patients were neutropenic (neutrophil count <0.5 G/L). The percentage of BSI in neutropenic patients (38%) was similar to that of non-neutropenic patients (35%). In both groups PCR/ESI-MS performed significantly better than BC (p = 0.013 in neutropenic and, p = 0.036 in non-neutropenic patients), whereas differences between SF and BC or between the molecular tests did not reach statistical significance. Finally, with regard to the infection source, in patients with pneumonia (n = 47) PCR/ESI-MS showed a significantly higher diagnostic capacity than SF (p = 0.020) and in particular BC (p < 0.001). In cases in which a central venous catheter was considered as the source of infection, both molecular tests performed considerably better than BC, but, due to the low number of cases (n = 14), the differences were not of statistical significance.

**Table 4:** Test performance in association with therapy, assigning ward and infection source.

patient subgroup		test performance characteristics (95% CI)	blood culture (BC)	BAC BSI	SeptiFast (SF)
antimicrobial therapy	no (n = 81, 35% BSI)	ROC-AUC	0.91 (0.82–0.96)	0.91 (0.83–0.96)	0.88 (0.78–0.94)
		% PPV	86.2 (70.7–94.2)	92.3 (75.3–97.9)	100 (–)
		% NPV	94.2 (84.8–97.9)	92.7 (83.7–96.9)	88.3 (79.9–93.5)
	yes (n = 112, 36% BSI)	ROC-AUC	0.67 (0.58–0.76)	0.89 (0.82–0.94)^a^	0.76 (0.67–0.83)
		% PPV	77.3 (57.5–89.5)	91.9 (78.8–97.2)	100 (–)
		% NPV	73.3 (67.8–78.2)	90.7 (83.2–95.0)	78.0 (72.2–82.9)
assigning ward	emergency room (n = 16, 63% BSI)	ROC-AUC	0.95 (0.72–1)	0.87 (0.61–0.98)	0.85 (0.59–0.98)
		% PPV	100 (−)	90.0 (59.8–98.2)	100 (−)
		% NPV	85.7 (48.3–97.5)	83.3 (42.9–97.1)	66.7 (43.7–83.8)
	intensive care unit (n = 25, 36% BSI)	ROC-AUC	0.60 (0.39–0.79)	0.94 (0.77–1)^b^	0.89 (0.70–0.98)^c^
		% PPV	60.0 (23.4–88.1)	100 (−)	100 (−)
		% NPV	70.0 (58.7–79.3)	94.1 (71.6–99.9)	88.9 (70.2–96.4)
	standard care (n = 87, 35% BSI)	ROC-AUC	0.81 (0.71–0.88)	0.89 (0.81–0.95)	0.80 (0.70–0.88)
		% PPV	87.0 (68.3–95.4)	89.3 (73.2–96.2)	100 (−)
		% NPV	84.4 (76.4–90.0)	91.5 (82.9–96.0)	82.6 (75.4–88.0)
	standard care oncology/hematology (n = 65, 29% BSI)	ROC-AUC	0.71 (0.58–0.81)	0.89 (0.79–0.95)^d^	0.75 (0.63–0.85)
		% PPV	71.4 (47.1–87.5)	94.1 (69.5–99.1)	100 (–)
		% NPV	80.4 (72.4–86.5)	91.7 (82.1–96.4)	81.8 (74.4–87.5)
infection source	Unknown (n = 96, 24% BSI)	ROC-AUC	0.79 (0.68–0.86)	0.87 (0.78–0.93)	0.81 (0.72–0.89)
		% PPV	75.9 (54.9–88.1)	94.7 (71.7–99.2)	100 (−)
		% NPV	88.2 (81.6–92.6)	92.2 (85.5–95.9)	88.9 (82.7–93.1)
	Respiratory tract (n = 47; 32% BSI)	ROC-AUC	0.57 (0.42–0.71)	0.95 (0.85–0.99)^e^	0.73 (0.58–0.85)
		% PPV	60.0 (21.8–89.0)	83.3 (63.0–93.6)	100 (−)
		% NPV	71.4 (65.7–76.6)	100 (–)	80.0 (71.4–86.5)
	Urinary tract (n = 19, 42% BSI)	ROC-AUC	0.83 (0.59–0.96)	0.83 (0.59–0.96)	0.75 (0.50–0.92)
		% PPV	85.7 (47.0–97.6)	85.7 (47.0–97.6)	100 (−)
		% NPV	83.3 (59.7–94.4)	83.3 (59.7–94.4)	73.3 (57.9–84.6)
	Central venous catheter (n = 14, 86% BSI)	ROC-AUC	0.63 (0.34–0.86)	0.92 (0.65–1.00)	0.88 (0.59–0.99)
		% PPV	90.0 (68.4–97.4)	100 (−)	100 (−)
		% NPV	25.0 (5.8–64.5)	50.0 (22.0–78.0)	40.0 (20.0–64.0)
	Others (n = 17, 59% BSI)	ROC-AUC	0.95 (0.73–0.99)	0.95 (0.73–1.00)	0.85 (0.60–0.97)
		% PPV	100 (−)	100 (−)	100 (−)
		% NPV	87.5 (52.2–97.8)	87.5 (52.2–97.8)	70.0 (47.5–85.7)

BSI = bloodstream infection according to the adapted European Centre for Disease Prevention and Control criteria; ROC-AUC = receiver operating characteristic-area under the curve; PPV = positive predictive value; NPV = negative predictive value; CI = confidence interval; ^a^BAC BSI vs. BC: p < 0.001 and BAC BSI vs. SF: p = 0.028, ^b^BAC BSI vs. BC: p = 0.007, ^c^SF vs. BC: p = 0.049, ^d^BAC BSI vs. BC: p = 0.020,^e^BAC BSI vs. BC: p < 0.001 and BAC BSI vs. SF: p = 0.020 as assessed by the Hanley & McNeil test.

## Discussion

The use of molecular tests allowing for the BC-independent pathogen detection in BSI facilitates rapid diagnosis and in a number of studies has been associated with higher diagnostic yield than BC^14,17,18,20,23,24,26,28,29^. Since the majority of the commercially available tests determine only a limited spectrum of common pathogens, the higher yield was shown, at least to some extent, to be associated with the prior use of antimicrobials and the resulting reduced viability of the causing agent in BC^15,30,31^. Theoretically, tests allowing for wide-range pathogen detection should exhibit an additional benefit over those with a limited panel particularly in patients of internal medicine wards and the emergency room, who may also be infected with less common fastidious or non-cultivable microorganisms. Until recently, the only commercially available broad-range molecular test in BC-independent BSI diagnostics was SepsiTest^TM^ (Molzym, Bremen, Germany). But using BC as reference, this test was shown to be less sensitive and specific than SF^32^. During the last years, PCR/ESI-MS has been considered a promising alternative for the direct, wide-range pathogen detection in blood and other usually sterile specimens^23–26^. The BAC BSI assay, which has recently been introduced for routine use, supported by the comprehensive database of the Iridica system should allow for the identification of all clinically relevant bacteria and yeasts. Furthermore, it allows for the identification and relative quantitation of multiple microorganisms in mixed infections, it detects the presence of other fungi - without being able to identify them - and its sensitivity may be comparable to that of SF as indicated by analytical evaluation data^33^. Considering these advantages, this test seemed to be most appropriate for the diagnosis of BSI in internal medicine wards and emergency room patients, which has now been confirmed by the results of this study.

As expected, one major advantage of PCR/ESI-MS over the other two tests proved to be the ability to detect microorganisms such as *Legionella pneumophila*, *Legionella longbeachae*, *Mycoplasma pneumoniae*, *Rickettsia typhi*, *Nocardia spp*., and fungi other than *Candida* spp., which are either non-cultivable or usually do not grow in BC and are also not included in the SF panel. As a matter of fact, with respect to pathogens included in the latter, no considerable difference between the two molecular tests was found, supporting the assumption of similar sensitivities on a pathogen level (Fig. 1). Furthermore, prior antimicrobial therapy was shown to be significantly associated with a better performance of PCR/ESI-MS particularly when compared to BC, which was attributable to a dramatic difference in sensitivity between the two tests in pre-treated patients (82.9% by PCR/ESI-MS vs. 41.5% by BC). This may reflect the influence of antimicrobials on the viability of potential pathogens resulting in a significantly higher positivity of a molecular method over BC as previously shown by others^31^. In contrast, in patients who were not pre-treated with antimicrobials the sensitivity of BC was, if only to a minor extent, even higher than that of PCR/ESI-MS (Table 4).

The antimicrobial pre-treatment rate in our ICU patients with 84% was shown to be exceptionally high. Thus, it was not surprising that in this group of patients PCR/ESI-MS performed significantly better than BC. However, this was also true for SF. In seven out of eight BSI cases the same pathogen was detected by both molecular tests, whereas in only two of those cases the identified microorganism was also grown in BC. In the single case, in which only PCR/ESI-MS was positive, the identified microorganism (*Nocardia* spp.) could not be detected by SF because it is not one of the pathogens included in the test’s panel. These data suggest that, perhaps due to a rather infrequent incidence of uncommon BSI pathogens in ICU patients, the use of a molecular method detecting a limited number of common pathogens may be nearly as sufficient as a wide-range pathogen detection test.

In accordance to published data^25,34^, the diagnostic yield of PCR/ESI-MS was to some extent lower in hematological/oncological patients than in those from the ICU (Table 4). However, also in onco-hematological patients, the significant difference in performance between PCR/ESI-MS and BC may be due to the increased antimicrobial treatment rate in these patients of 72.3%, which was considerably higher than that of the overall study population of 58%. The association between the application of antimicrobials and the better performance of the molecular tests, in particular PCR/ESI-MS, was evident also in the group of patients with pneumonia as source of BSI. Thus, 78.3% of these patients were under antimicrobial treatment. In contrast, in patients with urinary tract infection as source of BSI and in those from the emergency room, the fact that BC performed similarly or even better than the molecular tests was associated with low treatment rates of 31.6% and 37.5%, respectively. Due to the low number of cases in each group and the related risk of underestimation of ICU, oncology and hematology wards, or pneumonia as independent variables associated with a better performance of the molecular tests, we forbore from performing multivariate logistic regression analysis. For instance, in patients in whom the source of BSI was considered a central venous catheter infection, BC has underperformed due to loss of specificity rather than lower sensitivity as a result of antimicrobial treatment (Table 4).

In accordance to previous prospective and retrospective studies^19,29^, we observed a higher BSI rate in samples submitted from the emergency department compared to other wards. This may be explained by more apparent signs and symptoms of BSI in emergency room patients resulting in a more obvious indication for specific diagnostic measures. Due to the fact that most of these patients are naïve to antimicrobial treatment, which favors the performance of BC, they would rather profit from the short time-to-result of molecular testing rather than the higher sensitivity.

In this and recent studies^23–26,34^, PCR/ESI-MS technology and specifically the Iridica BAC BSI assay proved to be an enrichment in diagnostic laboratories. It stands to reason that BC is an indispensable test, as there are cases, in which pathogens are grown by BC whereas molecular tests are false negative; it also allows for a comprehensive susceptibility testing. However, molecular tests are a valuable add-on to culture and may increase the diagnostic yield in a considerable manner. BC combined with either BAC BSI or SF performed significantly better than BC alone (p < 0.001, Table 3). In particular, BC together with BAC BSI, as a wide-range pathogen detection complementary tool, achieved an excellent sensitivity of almost 96% and a negative predictive value above 97%. The fact that the lower cumulative specificity was due to cases of contamination, which were easily identified as such, emphasizes the high diagnostic accuracy of BC in combination with the BAC BSI assay.

Unfortunately, shortly after this study had been completed, the Iridica system was removed from the market. Presumably, this decision has a financial background considering the limited number of potential users and the high economical effort associated with the purchase and maintenance of the system, which proved to be interference-prone and service-intensive. One can only hope for a subsequent product with similar favorable characteristics in the near future.

In conclusion, in internal medicine patients with suspected BSI PCR/ESI-MS proved to be superior to SF and in particular to BC. This was essentially due to the ability of this technology for wide-range pathogen detection also including non-cultivable microorganisms and the weak performance of BC in patients with antimicrobial therapy. BC combined with PCR/ESI-MS showed a particularly high diagnostic accuracy. SF showed to be a reasonable addition to BC-based diagnostics, especially in the ICU, where most patients were pre-treated by antimicrobials and the identified microorganisms were rather common BSI pathogens which can be determined by the SF panel. Our data though, emphasize the need for both wide-range and highly sensitive molecular testing in BSI which may not be achievable by currently available molecular diagnostic tests.

## Methods

### Study design, specimen types and recorded data

This is a prospective, observational, monocentric study conducted at a tertiary care facility in consecutive patients being admitted to the emergency department or participating internal medicine wards - including intensive care units (ICUs) - with a clinically suspected BSI. For inclusion, patients had to fulfil at least two of the following SIRS criteria: body temperature >38 °C or <36 °C, heart rate >90 beat/min, tachypnea >20/min or PaCO2 < 32 mmHg, WBC count >12 000/mm^3^ or <4 000/mm^3^. Furthermore, the physician in charge should have prompted the collection of both blood cultures and an EDTA blood vial for SeptiFast PCR within the framework of routine microbiological diagnostics. Body temperature, heart rate, tachypnea or PaCO2 were considered at the time of the blood draw. In the great majority of cases, the sample for WBC count was collected at the same time and only in few cases within 24 h from the blood draw. Exclusion criteria were age under 18 years, refusal to provide written informed consent, participation in another study, HIV positive status, or surgery within the last 72 hours. All patients admitted from March 2015 to May 2016, who met the above criteria, were included in the study.

Two to three blood cultures each consisting of an aerobic (BacT/ALERT^®^ FA Plus) and an anaerobic bottle (BacT/ALERT^®^ FN Plus) were collected according to the guidelines of the German Society for Hygiene and Microbiology (DGHM). In addition, approximately 10 ml whole blood was drawn from the same venous puncture and collected into an ethylenediaminetetraacetic acid (EDTA) tube. EDTA whole blood was analyzed by PCR/ESI-MS and SF which has been performed routinely upon request as an add-on to BC diagnostics in BSI. The management of the patients included in the study was exclusively adapted to the results from the routine methods, namely BC and SF. The results from the non-routine test method (PCR/ESI-MS) were not disclosed, and therefore, had no influence on the antimicrobial therapy. Demographic, prospective and retrospective clinical and laboratory data including information regarding antimicrobial treatment and potential pathogens found in other specimen sites have been recorded of all patients in the study. Antibiotic treatment was considered relevant if it had been administered within 48 h before testing.

The performance of the two molecular tests was assessed in comparison to BC and that of each of the three tests in comparison to adapted ECDC criteria for laboratory confirmed BSI^27^. Thus, among our SIRS patients the criteria were as follows: (i) the detection of a recognised pathogen by one BC or any of the molecular tests, (ii) growth of a common skin contaminant in at least two BCs from separate blood samples within 48 h. The classification of infection cases other than BSI was done according to the definition of the ECDC, established for the point prevalence survey of health care-associated infections^27^. For patients who could not unambiguously be classified, a review committee including infectious diseases clinicians and microbiologists was established to classify each borderline case according to the majority of expert-votes.

### Ethical approval and informed consent

The study has been approved by the institutional review board of the Medical University Vienna. It has been performed in accordance with the ethical standards of the 1964 Declaration of Helsinki and its later amendments. Informed consent was obtained from all patients included in the study.

### Laboratory procedures

The blood culture bottles were incubated at 36.5–37 °C for up to 7 days in the semi-automated continuous-monitoring blood culture system BacT/ALERT 3D (BioMérieux, Marcy l′Etoile, France). Positive BC bottles were analyzed by Gram stain and the Sepsityper test using matrix-assisted laser desorption ionization-time of flight mass spectrometry (MALDI-TOF MS; Microflex; Bruker Daltonics, Bremen, Germany) followed by subcultures on appropriate solid media. Subculture isolates were again analyzed by MALDI-TOF MS to confirm the Sepsityper results and, if necessary, by other standard microbiological methods.

The EDTA whole blood sample for the molecular tests was processed according to the instructions of the respective manufacturer. SF consists of mechanical cell lysis and purification of DNA followed by three real-time amplification reactions for the detection of common Gram-positive, Gram-negative or fungal pathogens using hyprobes. This test allows for the detection of 20 relevant pathogens or groups of pathogens including the five most common *Candida* species and *Aspergillus fumigatus*. Coagulase-negative staphylococci (CoNS) and streptococci - with the exception of *S. pneumoniae* – can be identified at the genus level only. The most common *Klebsiella* species (*K. pneumoniae*, *K. variicola* and *K. oxytoca*) and *Enterobacter* species (*E. cloacae* and *E. aerogenes*) can all be detected but they cannot be identified to the species level. The Iridica BAC BSI assay (CE-IVD marked) combines DNA extraction, multiple broad-range amplification reactions of bacteria and *Candida* species DNA as well as amplification of four antibiotic resistance markers and finally analysis of amplification products by electrospray-ionization time-of-flight mass spectrometry (ESI-TOF MS). The system’s database contains approximately 800 entries each corresponding to a different microorganism. In positive cases, the amplicon profile of the unknown microorganism(s) in the sample was compared to the database entries by the system’s software resulting in pathogen identification.

The diagnostic findings by BC and SF were routinely evaluated by the respective physician in charge and were then communicated to the attending physician.

### Statistical analysis

The statistical analysis was performed using MedCalc (MedCalc Software bvba, Ostend, Belgium, Version 17.8). Metric data is shown as median (quartile 1–quartile 3) and group differences are analyzed by using the Mann-Whitney U test. Categorical data is presented as counts (percentage) and groups are compared by using the Chi-squared test. The predictive performance of BC, SF and PCR/ESI-MS was evaluated by using the area under the receiver-operating-characteristic curve (ROC-AUC). ROC-AUCs were compared by using the Hanley & McNeil test^35^. Confidence intervals were computed by using bootstrapping (in 1000 iterations). Statistical significance was defined as a p-value < 0.05 (two-sided). An accumulation of an alpha-error related to multiple testing was corrected by using the Bonferroni-Holm method.

## Electronic supplementary material

Dataset 1

## Data Availability

The datasets generated during this study can be made available by the corresponding author on reasonable request.
