# Supplementary material for: Substantial diagnostic impact of blood culture independent molecular methods in bloodstream infections: Superior performance of PCR/ESI-MS
Source: Sci Rep. 2018 Oct 30;8:16024. doi: 10.1038/s41598-018-34298-7 (PMC6207717; doi:10.1038/s41598-018-34298-7)
Supplement: Supplementary file 1 — Dataset 1 [file 41598_2018_34298_MOESM1_ESM.docx]

Substantial diagnostic impact of blood culture independent molecular methods in bloodstream infections: Superior performance of PCR/ESI-MS

Athanasios Makristathis^1^*, Nicole Harrison^2^, Franz Ratzinger^3^, Manuel Kussmann^2^, Brigitte Selitsch^1^, Christina Forstner^2,4^, Alexander M. Hirschl^1^ & Heinz Burgmann^2^

^1^Division of Clinical Microbiology, Department of Laboratory Medicine, Medical University Vienna, Vienna, Austria. ^2^Division of Infectious Diseases and Tropical Medicine, Department of Medicine I, Medical University Vienna, Vienna, Austria. ^3^Division of Medical-Chemical Laboratory Diagnostics, Department of Laboratory Medicine; Medical University Vienna, Vienna, Austria. ^4^Centre of Infectious Diseases and Infection Control, Jena University Hospital, Jena, Germany.

*Correspondence should be addressed to A.M. (athanasios.makristathis@meduniwien.ac.at)

**Supplementary Table S1.** Distribution of infections according to adapted European Centre for Disease Prevention and Control classification criteria, established for the point prevalence survey of healthcare-associated infections

| Type | ECDC class | n | % |
| --- | --- | --- | --- |
| Bloodstream infection^1^ | C-CVC^3^ (n=11), C-PVC^4^ (n=1), S-DIG^5^ (n=5), S-PUL^6^ (n=15), S-SST^7^ (n=1), S-UTI^8^ (n=8), S-OTH^9^ (n=4), S-UO^10^ (n=24) | 69 | 56.6% |
| Respiratory tract infection^2^ | PN3^11^ (n=2), PN4^12^ (n=2), PN5^13^ (n=27), LRI-Lung^14^(n=1) | 32 | 26.2% |
| Gastrointestinal system infection^2^ | GI-GIT^15^ (n=3), GI-IAB^16^ (n=1), EENT-ORAL^17^ (n=1) | 6 | 4.9% |
| Urinary tract infection^2^ | UTI-A^18^ (n=9), UTI-B^19^ (n=2) | 11 | 9.0% |
| Others^2^ | SYS-DI^20^ (n=1), SSI-S^21^ (n=1),  CRI1-CVC^22^(n=1), CRI2-CVC^23^(n=1) | 4 | 3.3% |
| Total |  | 121 | 100% |

^1^= potential pathogen detected by blood culture or any of the molecular tests ^2^= blood culture and both molecular tests negative, ^3^= blood stream infection (BSI), related-central vascular catheter; ^4^= blood stream infection (BSI), related-peripheral vascular catheter; ^5^= BSI, secondary digestive tract infection; ^6^= BSI, secondary-pulmonary infection; ^7^= BSI, secondary-skin and soft tissue infection; ^8^= BSI, secondary-urinary tract infection; ^9^= BSI, secondary-another infection; ^10^= BSI, (confirmed) unknown origin; ^11^=pneumonia, microbiological diagnosis by alternative microbiology methods, ^12^= pneumonia, positive sputum culture or non-quantitative culture from lower respiratory tract specimen; ^13^= pneumonia, clinical signs of pneumonia without positive microbiology; ^14^= lower respiratory tract infection, other than pneumonia; ^15^= GI, gastrointestinal tract infection (oesophagus, stomach, small and large bowel, and rectum) excluding gastroenteritis and appendicitis; ^16^= GI, intra-abdominal, not specified elsewhere; ^17^= eye, ear, nose or mouth infection (EENT), oral cavity (mouth, tongue, or gums); ^18^= urinary tract infection (UTI), microbiologically confirmed symptomatic UTI; ^19^= UTI, not microbiologically confirmed symptomatic UTI; ^20^= SYS, disseminated infection; ^21^= surgical site infection (SSI), superficial incisional, ^22^= local CVC-related infection (no positive blood culture); ^23^=CRI2-CVC: General CVC-related infection (no positive blood culture);
